# Response to Correspondence from Kolstoe and colleagues concerning our paper entitled, *Research approvals iceberg: How a ‘low-key’ study in England needed 89 professionals to approve it and how we can do better*

**DOI:** 10.1186/s12910-019-0433-3

**Published:** 2019-12-24

**Authors:** Mila Petrova, Stephen Barclay

**Affiliations:** Primary Care Unit, Department of Public Health and Primary Care, University of Cambridge, Institute of Public Health, Forvie Site, Robinson Way, Cambridge, CB2 0SR UK

**Keywords:** Ethics committees, Research [MeSH], Ethical review [MeSH], Ethics, Research [MeSH], Bioethics [MeSH], Institutional review boards, IRB, Biomedical ethics, Comment [publication type] [MeSH]

## Abstract

In their letter to the Editor in this issue, Kolstoe and Carpenter challenge a core aspect of our recently published case study of research approvals [BMC Medical Ethics 20:7] by arguing that we conflate research ethics with governance and funding processes. Amongst the key concerns of the authors are: 1) that our paper exemplifies a typical conflation of concepts such as governance, integrity and ethics, with significant consequences for claims around the responsibility and accountability of the organisations involved; 2) that, as a consequence of this conflation, we misrepresent the ethics review process, including in fundamental aspects such as the ethics approval-opinion distinction; 3) that it is difficult to see scope for greater integration of processes such as applying for funding, research approvals, Patient and Public Involvement, etc., as suggested by us. Here we present an alternative point of view towards the concerns raised.

Dear Editor,

Thank you for the opportunity to respond to the letter of Kolstoe and Carpenter [1] about our paper “Research approvals iceberg: how a ‘low-key’ study in England needed 89 professionals to approve it and how we can do better” [2]. We are grateful to our colleagues for the time and thought they have taken to write a formal response. We are also grateful to *BMC Medical Ethics* for providing a platform for debating an issue with profound, yet often neglected, consequences for our science and knowledge. We appear to share crucial common ground with the authors in agreeing that the ethics and governance processes are indispensable for good research, that they can be “laborious and highly frustrating”, and that we have a responsibility to improve them. Such agreement about the fundamentals will hopefully further the debate constructively, even if, most likely, we agree to disagree.

## Ethics **vs.** governance, or ethics **with** governance – depends on where you sit

The main point in our colleagues’ criticism is that “the paper contains a key misunderstanding by conflating different systems and processes”, namely around research ethics, on the one hand, and research governance, on the other. The authors note our acknowledgement of the distinction as “extremely important” but see our decision to consider the processes jointly as disappointing and “a significant mistake”. They also lay out helpfully for readers differences between research ethics and research governance, which we had only given in broad outlines.

This may be an occasion where the two sides agree on the facts, but not on how to combine and/or evaluate them. We agree fully with our colleagues that the processes have ‘slightly different philosophical and practical contribution to the conduct of “good science”’, perhaps less with their sharper subsequent rephrasing that “Research governance is related to research ethics, but operates under a different philosophy and using different methods”. We believe that the processes are significantly intertwined and interdependent even if, from certain perspectives, sufficiently different and distinct.

In our experience, governance approvals generally require a preceding favourable ethics committee opinion. The processes are not only intertwined but may become locked in a temporary Catch 22. For instance, the ethics committee may want to know where a study would be conducted, while the potential sites are asking for a favourable ethical opinion before committing to hosting it. Furthermore, the electronic IRAS system (Integrated Research Application System) enables both ethics review submissions and site-specific governance approvals and much of the study documentation, sponsor and researcher credentials submitted, within or outside IRAS, are identical.

Our main rationale for discussing the processes of ethics and governance approvals together related, however, to their place in the trajectory of a research study from a researcher’s perspective. These processes represent a distinct part of that trajectory, involving tasks and outcomes which are, to a significant degree, qualitatively different to those involved in applying for funding, collecting data, analysing data, writing up papers, etc. To us, this is a phase we can usefully call one of “study approvals” (we will return to the term “approval” below). We argue that most researchers are attuned to the broader similarities that define work within this phase rather than to fine, precise distinctions between the various components of it.

Of course, from the perspective of a representative of an ethics committee, or a sponsor, or a local R&D office, their work is very distinct from that done in “other” organisations. We accept that one of the unfortunate consequences of wanting to illustrate the whole that results from badly fitting parts is that efficient, even exemplary, parts of the system may be perceived to be just as “guilty” of the overall burden as the less efficient ones.

## Approval or opinion? One way or the other, we will do what it says

In addition to the choice of viewing ethics and governance approvals in tandem, our colleagues have challenged the use of the word “approvals” when it comes to the outcome of ethics review processes. They point out that the accurate term is “opinion”. The authors are completely right about the precise technical vocabulary (which we also remember from correspondence with ethics committees). However, we do not think that the standard connotations of the word “opinion” (or at least standard in a democratic world), such as sufficient flexibility in whether you follow it or not, apply in the context of research ethics committee opinions.

In the world of research, an ethics committee opinion is, for all intents and purposes, impossible to ignore. It is inconceivable in our day, age and part of the world to proceed with a study in spite of an unfavourable opinion by an ethics committee. This can only happen in secrecy, without any chance for the study findings being published in respectable journals, and with the researchers risking every consequence of being accused of scientific and/or ethical misconduct. We take the practical implications of an ethics committee opinion as justifying our use of the term “approvals”.

## It is complex enough, why make it extra complex?

The final point we will address concerns the critique of one of our proposals for “where next”, namely around bringing closer together ethics and governance approvals and aspects of processes around grant applications and reporting; PPI (Patient and Public Involvement); dissemination, impact and public engagement and various other outward-facing research activities. Our colleagues have perceived this proposal as ignoring the distinctiveness of phases in research and exacerbating the conflation they have tried to illuminate. To clarify the latter point, we have in no way included those processes in our estimates of approvals burden. We argued that they have areas of overlapping concerns and documentation and that there is value in their closer integration in the future.

Our proposal is for a dynamic outward-facing profile of a research study which grows and changes with time, is accessed by different stakeholders at different stages of the work, enables efficient documentation review and sharing, and supports the goals of transparency and accountability. It is beyond the scope of this brief letter to expand on such a vision. Its success or otherwise is also an empirical rather than theoretical matter, and we hope to see prototype platforms in the not too distant future.

## Eternal vigilance

There is a saying that the price of a metaphor is eternal vigilance, which makes us offer the following one cautiously. The position of a health research study relative to the multiple stakeholders granting it the green light to proceed/ enabling its progress is similar to that of a child under the care of multiple providers.

As researchers, we are perhaps like the parents of a child needing routine healthcare, fearful of its well-being, feeling its impact on our well-being, and (over-)sensitive to the lack of clear pathways and the failure of communication and coordination between services.

As people who, for the purposes of this debate, inhabit mostly their roles of research ethics committee chairs, the authors are perhaps like healthcare providers in one of the many teams caring for the child, trying hard to support both the patient and us, and (over-)sensitive to the value of clear boundaries and the associated allocation of responsibility and accountability.

The tension will always be there, but we hope that this response represents an opportunity to reduce it in positive and productive ways.

## Data Availability

The datasets generated and analysed for the purposes of the study being debated are not publicly available, as a significant proportion of them identifies people and organisations, but anonymised subsets of them are available from the corresponding author on reasonable request.
